# Male choriocarcinoma presenting with intracerebral hemorrhage: a case report and literature review

**DOI:** 10.3389/fonc.2025.1549261

**Published:** 2025-04-10

**Authors:** Jie Chen, Yu He, Junwen Ao, Zhengshi Wang, Kuan Yang, Miao Chen, Xing Zhao

**Affiliations:** ^1^ Department of Critical Care Medicine, Kweichow Moutai Hospital, Zunyi, China; ^2^ Department of Clinical Laboratory, Affiliated Hospital of Guilin Medical University, Guilin, China; ^3^ Department of Pathology, Kweichow Moutai Hospital, Zunyi, China; ^4^ Department of Neurosurgery, Kweichow Moutai Hospital, Zunyi, China

**Keywords:** choriocarcinoma, intracerebral hemorrhage, testis, tumor metastasis, β-HCG

## Abstract

Male choriocarcinoma is an extremely rare malignant tumor. It has the characteristics of hidden onset, easy invasion and metastasis. Clinically, when choriocarcinoma is found in a patient, most of the tumor cells have metastasized to distant organs. This case reports a 65-year-old man with choriocarcinoma with two intracerebral hemorrhages as the main symptom. In this case, we consider the origin of intracranial choriocarcinoma to be testicular. We analyzed the clinical data of this patient and summarized the cases of male choriocarcinoma reported in the past, so as to arouse the attention of clinicians.

## Introduction

Choriocarcinoma is a highly aggressive malignancy. The disease is extremely rare, especially in men (1). Male testicular choriocarcinoma accounts for less than 1% (0.19%) of testicular germ cell tumors, and its annual incidence is (2-4)/10 million, accounting for only 0.01%~0.02% of male malignant tumors (2). At present, there are few reports about choriocarcinoma of testis. There is still a lack of effective early diagnostic methods for Testicular choriocarcinoma. The vast majority of testicular choriocarcinoma are found to have metastasized far away. The main treatment for it is surgical resection combined with chemotherapy, but the prognosis is poor (3). This study reports the case of a 65-year-old man with choriocarcinoma, which presented primarily with two intracerebral hemorrhages. According to the patient’s medical history and relevant examination results, it was considered that the intracranial metastasis of testicular choriocarcinoma occurred and caused intracerebral hemorrhage by destroying intracranial blood vessels. At the same time, we reviewed the relevant literature in order to improve the understanding of intracranial metastasis of choriocarcinoma of testis.

## Case report

**Figure 1:**
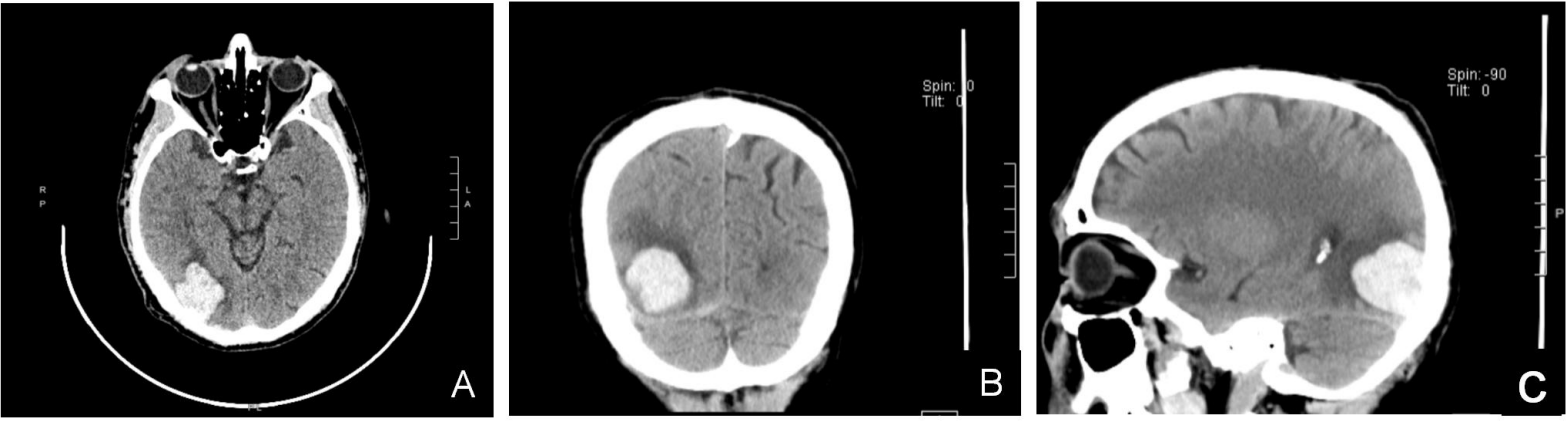
**(A–C)** Head CT scan (plain scan + 3D reconstruction): A flake high-density shadow was seen in the right occipital lobe with a CT value of about 70HU, with clear boundaries and low density shadows around it.

A 65-year-old male patient was admitted to hospital with the main complaint of sudden right limb weakness for 1 hour. The patient simultaneously experienced symptoms of headache and dizziness. The patient was unable to walk due to weakness of the right limb and was admitted in a wheelchair. His headaches and dizziness were tolerable. He has a history of hypertension for 6 years, and his blood pressure is well controlled. He had recurrent right scrotal pain and discomfort in the past 6 months, but he did not pay attention to it. Two months before this admission, the patient had been admitted to another hospital with headaches. The patient was conscious and had symptoms of nausea. There were no symptoms of blurred speech, numbness or weakness in the limbs. The physical examination indicated a neurological Glasgow score of 15. His limb strength was level 5, physiological reflexes were normal, and pathological signs were negative. A computed tomography (CT) scan of the head revealed a cerebral hemorrhage in the right occipital lobe of the brain (**Figures 1A–C**). Chest CT showed no abnormalities in both lungs. Electrocardiogram and echocardiography showed no significant abnormality. Head CT examination showed right occipital cerebral hemorrhage, and the patient had mild symptoms, so he was unwilling to undergo further MRI examination. Due to the small amount of intracranial bleeding, no surgical treatment was performed. During hospitalization, the main treatments included 125ml mannitol given intravenously 3 times a day for dehydration for 4 days, 1g tranexamic acid given intravenously once a day for hemostasis for 3 days, and nifedipine sustained release tablet 20mg once a day for hypertension. After 5 days in hospital, head CT indicated that the cerebral hemorrhage had been partially absorbed. After 8 days in the hospital, the patient had almost no headache symptoms, so he was discharged. The patient denied any history of fall injury and family history of intracerebral hemorrhage.

**Figure 2:**
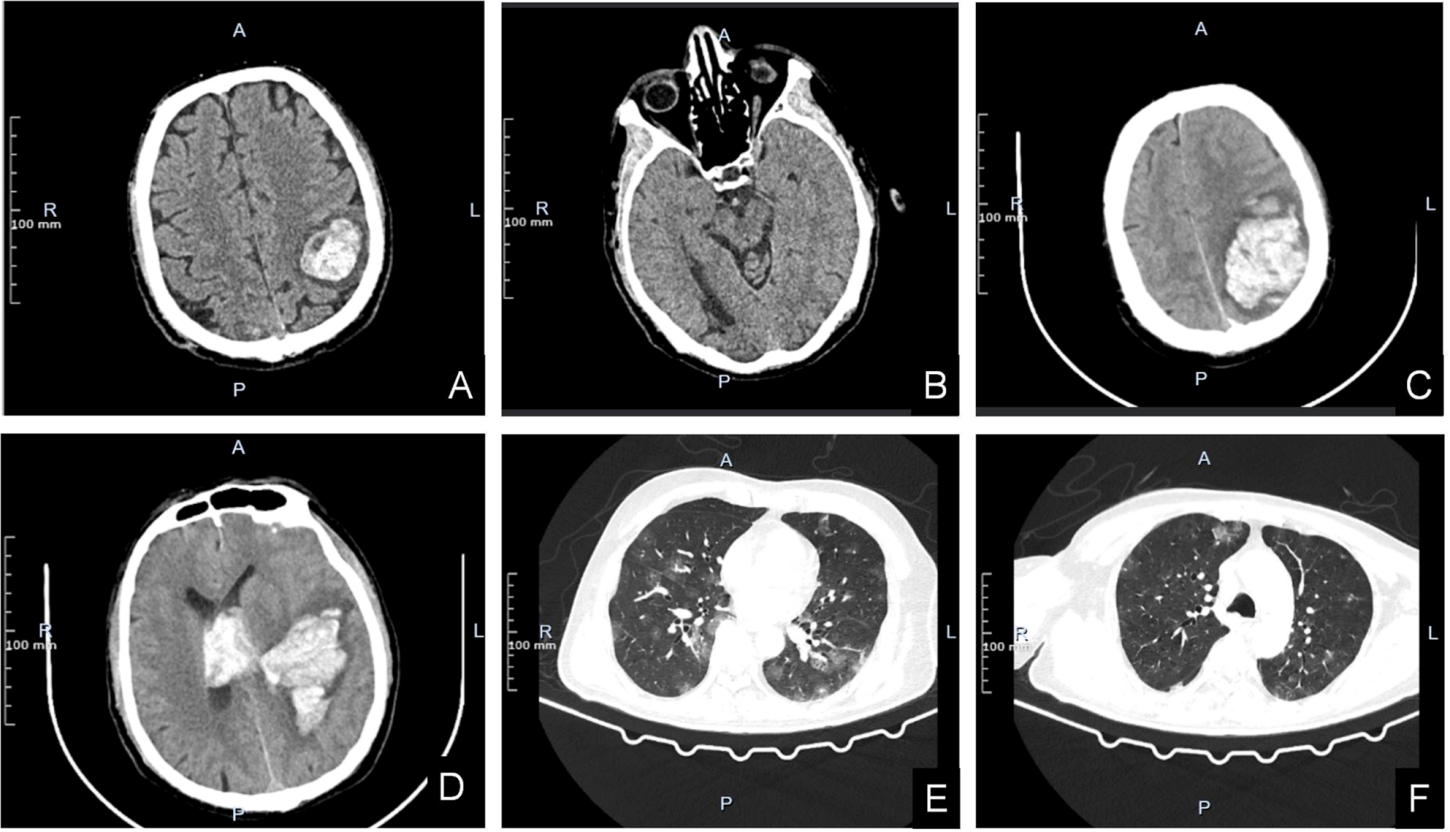
**(A, B)** Head CT scan on admission. The left parietal lobe has A dense mass with mild peripheral edema **(A)**. The right occipital lobe has a patchy low-density shadow with poorly defined boundaries **(B)**. **(C–F)** Head and chest CT scans during hospitalization when disease worsens. High-density mass shadows are seen in the left parietal and occipital lobes, surrounded by low-density shadows. The linear structures in the brain are compressed and shifted to the right **(C)**. Left parietal lobe dense mass shadow penetrated into the ventricle. The cast high density shadow was seen in the bilateral lateral ventricles, the third and fourth ventricles **(D)**. Lung CT scan revealed multiple metastases in both lungs with unclear boundaries **(E, F)**.

**Figure 3:**
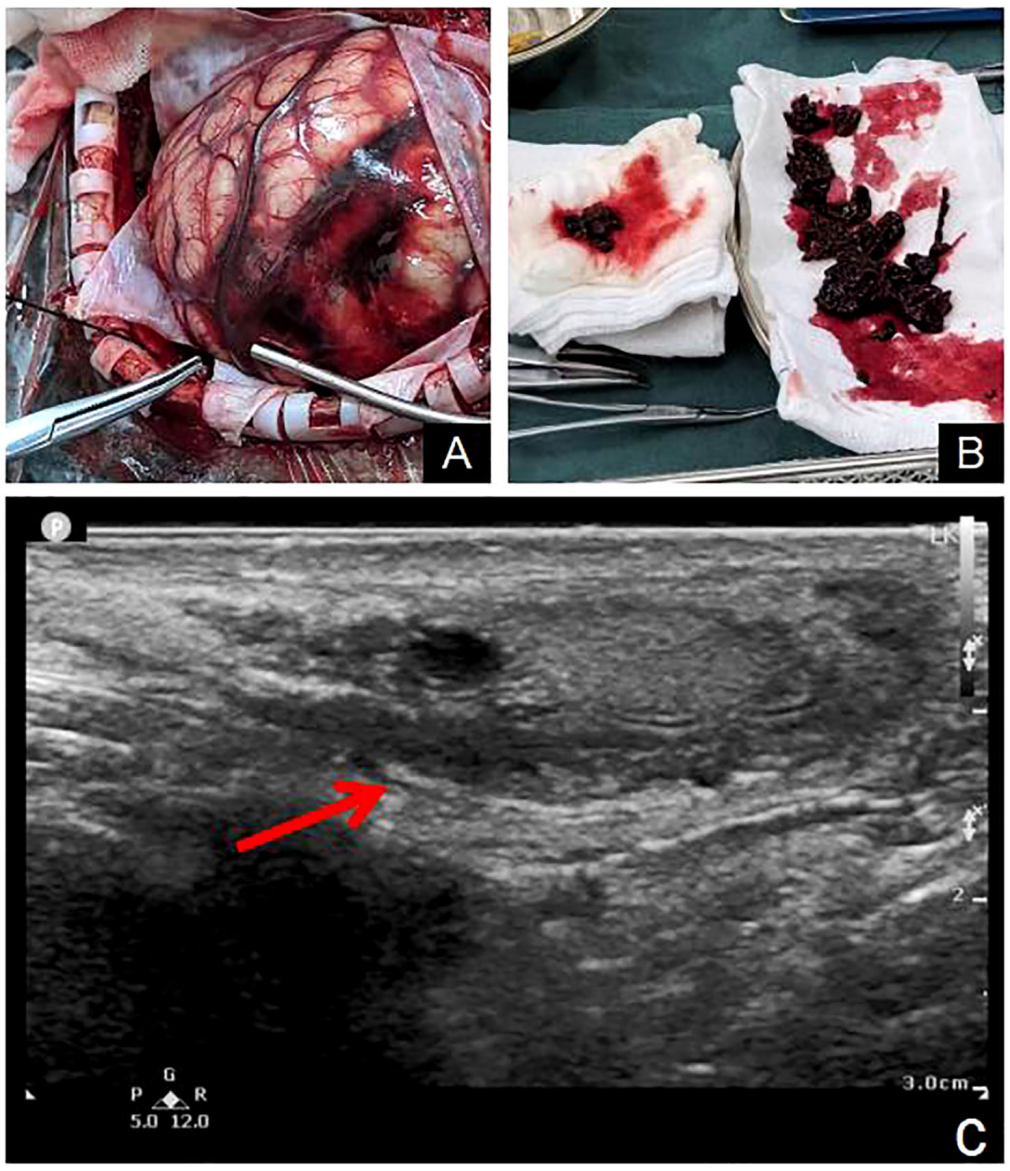
Arteriovenous malformations of the left parietal and occipital lobes were observed during the operation **(A)**. The vessel capsule was resected and emboli was found in the vessel **(B)**. Scrotal ultrasound revealed mixed echo masses in the right testicle **(C)**.

At the time of this admission, the physical examination indicated that the body temperature was 36.5°C, the heart rate was 80 beats/min, the respiration was 20 beats/min, the blood pressure was 165/84mmHg, and the blood oxygen saturation was 99%. He was conscious and he could answer questions correctly. Cardiopulmonary and abdominal examination showed no abnormality. He had a Glasgow score of 15. Both pupils were 3mm in diameter and sensitive to light reflection. The right limb muscle strength was level 4, the left was level 5. Meningeal stimulation was negative. The muscular tone of the extremities was not elevated, the physiological reflexes were normal, and the Babinski sign and Kernig sign were negative. The right testicle had a 1×2cm regular mass without tenderness. Rest of the physical examination showed no obvious abnormality. On admission, head CT indicated cerebral hemorrhage in the left parietal lobe, and previous hemorrhage in the right occipital lobe had been absorbed, but cerebral infarction was complicated (**Figures 2A, B**). His laboratory tests such as blood routine, liver and kidney function, coagulation function, thyroid function, infectious disease series, electrolytes were normal. After admission, the patient’s headache symptoms gradually worsened. An hour later, the patient suddenly fell unconscious. Both pupils were 3mm in diameter, and their sensitivity to light reflection became insensitive. Emergency head CT examination revealed significant aggravation of intracerebral hemorrhage, deviation of cerebral line, and cerebral hernia (**Figures 2C, D**). Chest CT showed multiple nodules in both lungs (**Figures 2E, F**). The neurosurgeon immediately performed an emergency craniotomy, during which a left parieto-occipital arteriovenous malformation, blood vessel rupture, and subarachnoid hemorrhage were observed (**Figure 3A**). About 100ml of hematoma was removed. Embolus were evident in malformed arteriovenous blood tubes (**Figure 3B**).

**Figure 4:**
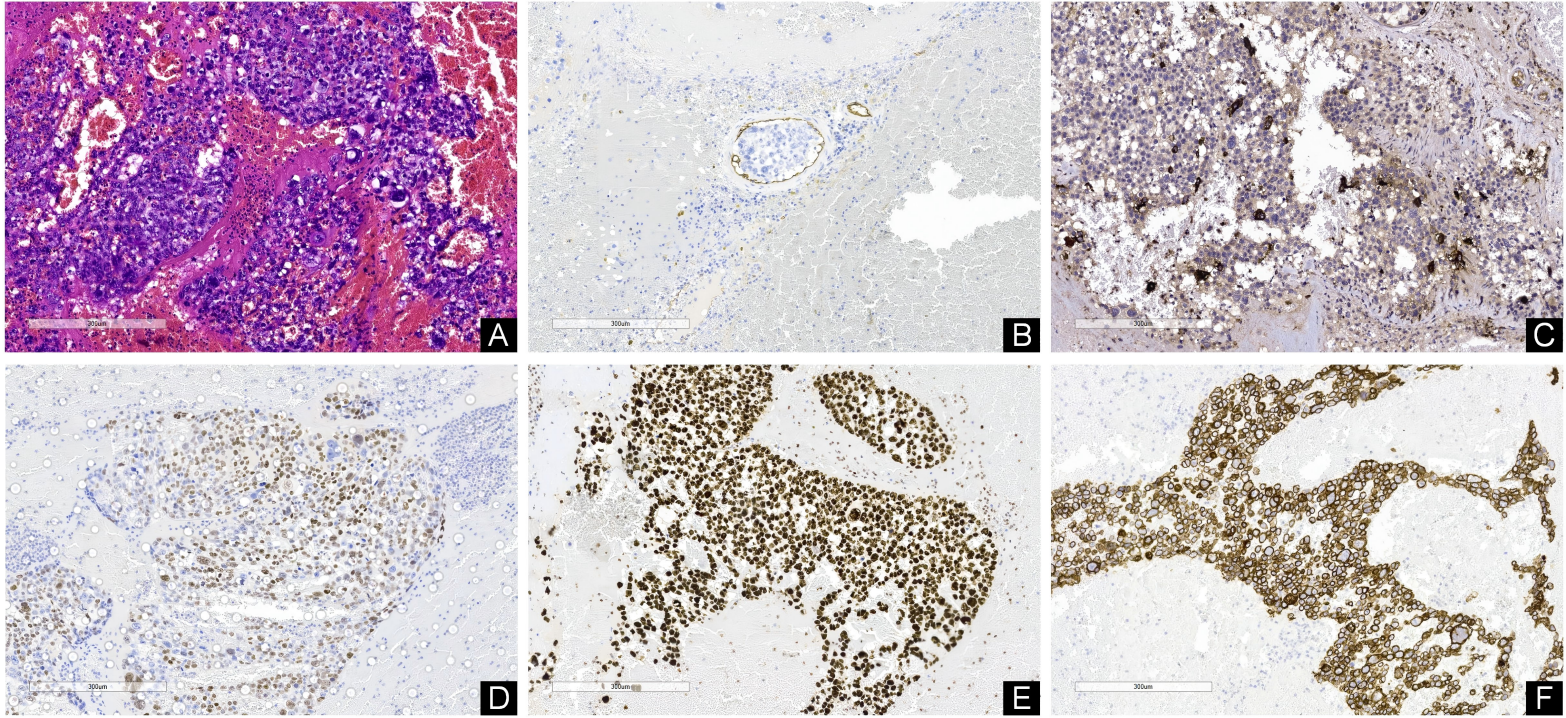
**(A)** Hematoxylin-eosine-stained sections revealed cells with hyperchromatic, pleomorphic nuclei; prominent nucleoli; and increased mitotic activity. **(B)** Immunohistochemistry indicated the presence of cancer thrombus. Immunohistochemical staining showed that β-HCG **(C)**, P63 **(D)**, Ki67 **(E)** and CK **(F)** were all positive. [Original magnifications: **(A–F)** 100x].

The resected vascular capsule and intravascular embolus were sent for pathological examination. After the operation, he was transferred to the intensive care Department for further care and treatment. Acute Physiology and Chronic Health Evaluation II had a score of 26. He was kept on a ventilator while he was in intensive care. Remifentanil combined with midazolam was injected intravenously for sedation and analgesia, and the Richmond Agitation-Sedation Scale fluctuated between -4 and -2 points. Urapidil was continuously pumped intravenously to control blood pressure, which fluctuated between 130-140/70-80mmHg. For the first 3 days after surgery, 60mg/h of sodium valproate was injected intravenously to prevent seizures. For the first 2 days after surgery, tranexamic acid 1g once a day was given intravenously to stop bleeding. On the second day after surgery, the patient’s temperature increased to 38.5°C. White sputum could be sucked out of the tracheal tube. A physical examination of both lungs suggested moist crackles. The WBC count was 12.86×10^9^/L, the percentage of neutrophil was 94.6%, the calcitonin was 4.93ng/ml, and the absolute value of neutrophil was 12.17×10^9^/L. The hypersensitive C-reactive protein was 195.38mg/L. Chest CT examination revealed double lung pneumonia. Considering the diagnosis of severe pneumonia, meropenem 1g was given intravenously every 8 hours for anti-infective therapy. On the fourth day after surgery, the patient’s sputum culture results indicated Klebsiella pneumoniae, which was sensitive to meropenem. After 24 hours postoperatively, 125ml mannitol was administered intravenously every 8 hours for dehydration. On the 3rd day after surgery, head CT showed that brain edema was serious, so 125ml mannitol was adjusted every 6 hours to enhance dehydration and reduce intracranial pressure. On the 7th day after surgery, head CT indicated that the cerebral edema had decreased, so the 125ml mannitol was adjusted again every 8 hours. Percutaneous tracheotomy was performed on the 4th postoperative day. In addition, he received treatments such as maintaining electrolyte balance, Fluid infusion, and nutritional support. On the fifth day after the operation, sedation was discontinued and the patient’s consciousness returned to a light coma. For the patient’s right testicular mass, color ultrasound examination of the scrotum indicated a testicular germ-cell tumor (**Figure 3C**). Neuron-specific enolase, squamous cell carcinoma antigen, carcinoembryonic antigen, carbohydrate antigen 199, prostate specific antigen, and alpha-fetoprotein were all in the normal range. However, beta-human chorionic gonadotropin (β-HCG) was significantly elevated, reaching 20,326 mlU/ml. On the 10th day after surgery, hematoxylin-eosin staining and immunohistochemical results confirmed choriocarcinoma (**Figures 4A–F**). Immunohistochemistry of tumor cells showed: CD31 (-), CD34 (-), Ki67(Li: 95%), HMB45 (-), MelanA (-), S - 100 (-), CK (+), Vimentin (-), CD79a (-), CD20 (-), CD3 (-), EBV (-), CD10 (-), HPL (-), about (+), the Bcl-6 (-), Bcl-2(-), CD30(-), Cmyc(-), P53 (wild type), MUM1 (-), CyclinD1 (-), PLAP (focal +), PSAP (-), CD117 (-), 0CT3/4 (-), SALL4 (-), CK7 (+), CK20(-), EMA (Focal point +), TTF-1 (-), PSA (-). The urological consultation indicated that the patient was in critical condition and could not be treated surgically. If the patient’s condition improves, orchiectomy is feasible to treat the testicular lesion. On the 10th day after surgery, the patient was transferred to a local hospital to continue receiving ventilator assisted ventilation, anti-infection, nutritional support and other treatments. The patient died two months later due to severe lung infection and multiple organ failure.

## Discussion

We analyzed 10 cases of intracranial hemorrhage caused by metastatic choriocarcinoma of testis reported in Pubmed database. We also included the patients reported in this case (**Table 1**). We found patients between the ages of 17 and 65. Most patients complained of more than two symptoms, most of which included headache (8/11, 73%). Other initial or combined symptoms included decreased level of consciousness (3/11, 27%), Seizures (2), changes in limb muscle strength (2), limb paraesthesia (1), scrotal pain (1), and hemianopsia (1). β-HCG was detected in 6 of all patients and was significantly elevated (range: 18,339-513,389 IU/L). Four patients presented with multiple intracranial hemorrhage, the main bleeding sites included parietal (6), frontal (4), occipital (3), cerebellar (1), intracerebral supratentorial (1). Almost all patients had lung metastases (10/11, 91%). Other sites of metastasis included skin (1), kidneys (1), gastrointestinal tract (2), mediastinum (2), liver (2), retroperitoneum (2), pancreas (1), bone (1), thyroid (1). The majority of patients underwent surgery (10/11, 82%), with 3 patients receiving chemotherapy after surgery. In addition, three patients received chemotherapy alone. The overall prognosis was poor, with most patients dying during treatment (7/11, 64%) and the remainder in remission during follow-up.

**Table 1:** Clinical data of intracranial hemorrhage caused by metastatic testicular choriocarcinoma.

Case	Year	Age	Presentation	β-HCG (IU/L)	Hemorrhagic Site	Additional Sites	Treatment	Follow-up
1 (4)	2023	34	Seizures, headache	185,667	Parietal	Lung, liver, gastrointestinal tract, retroperitoneum	VIP	Death
2 (5)	2021	23	Headache	513,389	Intracerebral supratentorial	Lung	Craniotomy+ VIP	Live
3 (6)	2017	24	Seizures, headache	18,339	Cerebellar	Lung, kidney, retroperitoneum	Craniotomy+BEP	Live
4 (7)	2015	19	Headache	–	Frontal	Lung	Craniotomy	Death
5 (8)	2015	26	Headache, homonymous hemianopsia	–	Occipital	Sternum	Craniotomy	Live
6 (9)	2015	18	Altered mental status, headache	355,000	Frontal, parietal	Lung, mediastinum	Craniotomy+BEP	Death
7 (10)	2011	20	Loss of consciousness	–	Parietal, occipital	Lung, liver	Craniotomy	Death
8 (11)	2009	17	Headache, paresthesia	192,575	Frontal, parietal	Lung, skin, thyroid, mediastinum, pancreas	PEI	Live
9 (12)	1991	21	Limb paralysis	–	Parietal	Lung	Craniotomy	Death
10 (12)	1991	24	Semicomatose	–	Frontal	Lung, gastrointestinal tract	Craniotomy	Death
11	This case	65	Limb weakness, headache, scrotal pain,	20,326	Occipital, parietal	Lung	Craniotomy	Death

VIP, cisplatin, etoposide, and ifosfamide; BEP, bleomycin, etoposide, cisplatin; -, no data in the literature; PEI, cisplatin, etoposide, ifosfamide.

Choriocarcinoma mainly exists in two forms: gestational and non-gestational. Non-gestational choriocarcinoma originates from germ cells outside the gestational environment and is often found in the ovaries, testicles and mediastinum (13). Choriocarcinoma of the testis is a kind of non-seminoma germ cell tumor, which is the rarest in the testis, accounting for about 1-3% of all testicular tumors (14). At present, the cause of choriocarcinoma of testis is not completely clear. According to epidemiological analysis, there are many risk factors, among which cryptorchidism is one of the most important factors found so far. It has been reported in literature that the probability of choriocarcinoma in patients with cryptorchidism is 20 to 40 times higher than that in normal patients (15). In addition to the testicle itself, it may also be related to family genetic factors, gonadal hypoplasia, endocrine disorders, etc. Genetic studies have shown that testicular tumor is related to short arm ectopia of chromosome 12, and the change of p53 gene is also closely related to the occurrence of testicular tumor (16). The patients we reported had no previous history of cryptorchidism and no family genetic factors, so the specific mechanism was not clear.

Choriocarcinoma is a highly aggressive and rapidly progressing malignancy. Early rapid hematogenous spread to distant organs is one of the most important biological behaviors of choriocarcinoma. Therefore, at the time of diagnosis, the vast majority of cases have already metastasized, and metastatic site symptoms have already appeared. Due to the lesion’s strong ability to invade blood vessels, patients often have symptoms of bleeding at the site of metastasis (17). The most common sites of distant metastasis of choriocarcinoma are lung, liver and brain (18). About 10% of choriocarcinoma has intracranial metastasis (19). In addition, there are also some uncommon organs such as skin, stomach, and small intestine (20). When metastasized to the brain, patients often present with headache, fainting, and stroke-like symptoms (18). When cancer cells metastasize to the digestive tract, the main symptoms of patients are black stool and/or hematemesis and anemia (17). In our case, the patient had two episodes of intracranial hemorrhage within a short period of time, manifested as headache symptoms.

The main cause of intracerebral hemorrhage caused by brain metastasis of choriocarcinoma is the invasion of blood vessels by cancer cells, which is often fatal to patients. Specific pathogenesis may include vascular obstruction caused by tumor embolization, proliferation of tumor cells in the blood vessel wall, rupture of the elastic layer inside the blood vessel, and the resulting aneurysm (21). Choriocarcinoma invades the vascular wall due to the syncytiotrophoblastic component of the tumor, which has the innate ability to invade the endothelium and expand into the perivascular space (8). Malformations can result when blood vessels are invaded. It may also be that the original vascular malformation and choriocarcinoma metastasis exist simultaneously (6). In our case, the patient had two intracerebral bleeds two months apart. The first intracranial hemorrhage was occipital lobe hemorrhage, and the amount of bleeding was small. The second intracerebral hemorrhage is very large and required immediate surgery to remove the hematoma and relieve intracranial pressure. Vascular malformations and embolus formation were observed during the operation. The pathological findings of the malformed vessels suggest invasion of chorionic cancer cells.

Metastatic choriocarcinoma is difficult to identify at first diagnosis. It is usually confirmed by pathology of the intraoperative specimen. Considering that choriocarcinoma may lead to vascular malformations, MR angiogram may make it difficult to distinguish bleeding due to primary vascular malformations from bleeding due to choriocarcinoma (22). Angiography usually does not show lesions in the absence of acute bleeding, but can indicate the presence of vascular malformations (6). Positron emission tomography scanning may be valuable in detecting occult metastases of choriocarcinoma (23). The typical histopathological feature of metastatic choriocarcinoma is the coexistence of cytotrophoblastic and syncytio-trophoblastic cells, which is different from other germ cell tumors with syncytio-trophoblastic cells only. Serum β-HCG can be used as a characteristic tumor marker in the diagnosis of choriocarcinoma, and it is significantly elevated in patients with choriocarcinoma (24). A literature review by Yokoi et al. reported that 96.4% of choriocarcinoma patients had abnormally elevated serum β-HCG levels (25). Elevated alpha-fetoprotein suggests the presence of other histological components in choriocarcinoma, such as embryos, teratomas, or yolk sacs. In our case, the patient had right scrotal swelling for 6 months. Unfortunately, the clinician did not complete the β-HCG test when the patient was hospitalized for the first intracerebral hemorrhage. After the patient’s condition was stable, angiography was not completed to evaluate the intracranial blood vessels. During this hospitalization, the patient’s right testicular mass could be detected by physical examination. Further ultrasound examination of the scrotum indicated germ cell tumor. Skull CT did not indicate tumor occupation, and vascular malformations and intravascular emboli were observed during the operation. On admission 2 months ago, CT results showed no metastases in both lungs. However, in this hospitalization, chest CT indicated that pulmonary metastasis had occurred. According to the patient’s serum β-HCG was significantly elevated. At the same time, the pathological results of the operative specimens showed cytotrophoblast and syncytio-trophoblast. Therefore, we consider the occurrence of intracranial and pulmonary metastasis of testicular choriocarcinoma, the invasion of intracranial blood vessels by cancer cells to form vascular malformations, and eventually lead to vascular rupture and bleeding.

Choriocarcinoma usually metastasizes extensively in the absence of obvious manifestations of the primary tumor, resulting in a poor prognosis. For early stage non-metastatic choriocarcinoma of testis, it is possible to obtain long-term tumor free survival or even cure with orchiectomy of the diseased side combined with chemotherapy. For metastatic choriocarcinoma, individualized treatment should be performed according to the metastatic situation (26). For choriocarcinoma of testis with metastasis, comprehensive treatment measures, mainly chemotherapy, are usually adopted (14).

With the continuous improvement of various treatment methods, choriocarcinoma can be significantly relieved after standardized treatment. However, for patients with brain metastases, the prognosis is still poor (27). When acute massive intracerebral hemorrhage occurs in patients with intracerebral metastasis, the mortality rate is extremely high (28). According to literature statistics, 8 out of 9 such patients died (6). Patients often die from brain hernias. Therefore, surgical treatment is urgently needed to remove intracranial compression to save lives and create opportunities for later chemotherapy (27). Haydn et al. (6) reported a case of brain metastasization of choriocarcinoma with no bleeding or acute mass effect. The patient underwent complete tumor resection and then received chemotherapy with bleomycin, etoposide and cisplatin, which achieved satisfactory results. Similarly, Sundarakumar et al. (8) reported a case of arteriovenous malformation caused by metastasis of choriocarcinoma, which achieved good results after complete tumor resection. Therefore, in the absence of serious complications in metastatic diseases, surgical intervention is very necessary (6, 29). Yang et al. (27) believed that for patients with intracranial hypertension and cerebral hernia, treatment should not be abandoned because the patient’s life is in danger. However, active surgery and chemotherapy will significantly improve the prognosis of patients and improve the survival rate of patients with advanced choriocarcinoma brain metastases. In conclusion, regardless of the presence or absence of critical symptoms due to tumor metastasis, aggressive neurosurgical treatment is initiated so that patients have the opportunity to receive further chemotherapy regimens to prolong survival. At present, patients with choriocarcinoma brain tissue metastasis often die in a short period of time, and there is a lack of long follow-up time. Therefore, there is no effective conclusion on the long-term prognosis after tumor resection. In addition, Guo et al. (30) reported a case of intracranial choriocarcinoma treated with radiotherapy, and the patient survived for ~6 months. However, there are few reports on the efficacy of radiotherapy for distant metastatic choriocarcinoma. Stereotactic radiosurgery (SRS) is a non-invasive treatment modality that has been used in recent years to treat various intracranial tumors. Kohyama et al. (31) reported the effectiveness of SRS in the treatment of primary intracranial choriocarcinoma in 2001. SRS is a viable method to treat intracranial metastasis of choriocarcinoma, especially in cases where immediate surgical treatment is not required (32). Therefore, SRS may be a feasible approach for patients with brain metastases of choriocarcinoma without intracranial hemorrhage. In our case, the patient was in life-threatening condition due to a large amount of intracranial bleeding, so an emergency operation was performed to remove intracranial hematoma and reduce intracranial pressure to save his life. Complications such as severe pneumonia and respiratory failure occurred after operation, and his condition was extremely critical. The family considered that the patient’s survival time might be short, so they refused chemotherapy treatment. The patient died on the 70th day after surgery.

At present, there is little literature on the clinical course, diagnosis and treatment of pure choriocarcinoma brain metastases in men. This makes it easy for clinicians to overlook the presence of the disease in the diagnosis and treatment of intracerebral hemorrhage. In addition, omitting physical examinations and laboratory tests will lead to misdiagnosis of the disease. In this patient, the testicular tumor was not detected due to the lack of careful testicular physical examination, scrotal ultrasound and serum β-HCG. By the time we discovered the presence of choriocarcinoma, the patient’s disease had advanced to a serious stage. The patient survived only 70 days due to second intracerebral hemorrhage and associated severe complications.

## Conclusion

Male choriocarcinoma is a rare malignant tumor with high invasive and early hematologic metastasis, which seriously threatens the life and health of patients. In order to prolong the survival time of patients and reduce the medical burden, early diagnosis is particularly important. The significance of this case is that serum β-HCG testing is very important for unexplained bleeding at any site.

## Data Availability

The original contributions presented in the study are included in the article/supplementary material. Further inquiries can be directed to the corresponding authors.

## References

[1] Kyriakou F Vaslamatzis MM Bastani S Lianou MA Vourlakou C Koutsoukou A . Primary choriocarcinoma of the renal pelvis presenting as intracerebral hemorrhage: a case report and review of the literature. J Med Case Rep. (2011) 5:1–4. doi: 10.1186/1752-1947-5-501 21975326 PMC3197539

[2] Chhieng DC Jennings TA Slominski A Mihm MC Jr . Choriocarcinoma presenting as a cutaneous metastasis. J Cutan Pathol. (1995) 22:374–7. doi: 10.1111/j.1600-0560.1995.tb01423.x 7499580

[3] Wei H Zhang T Liu B Xue X Wang G . Choriocarcinoma of unknown origin with multiple organ metastasis and cerebral hemorrhage: A case report and literature review. Oncol Lett. (2016) 11:3749–52. doi: 10.3892/ol.2016.4463 PMC4888274 27313687

[4] Le DP Hall SC . Medical literature writing with ChatGPT: A rare case of choriocarcinoma syndrome with hemorrhagic brain metastases due to burned out metastatic mixed testicular cancer. Cureus. (2023) 15:e36655. doi: 10.7759/cureus.36655 37009366 PMC10065126

[5] Aftan MK Badrawi N Abdulghaffar S Abedzadeh AA Albastaki U Raman LG . Pure testicular choriocarcinoma Cannonball metastases as a presenting imaging feature: A case report and a review of literature. Radiol Case Rep. (2021) 16:923–8. doi: 10.1016/j.radcr.2021.01.053 PMC7881168 33613805

[6] Hoffman HA Toshkezi G Fullmer JM Hall W Chin LS . Pitfalls in diagnosis and management of testicular choriocarcinoma metastatic to the brain: report of 2 cases and review of literature. World Neurosurg. (2017) 106:536–42. doi: 10.1016/j.wneu.2017.07.023 28712904

[7] Morollón N Arrese I Zamora T Sarabia R . Histology of a cerebral hemorrhage: AVM as a seat of a metastatic choriocarcinoma. Neurocirugia (Astur). (2015) 26:143–6. doi: 10.1016/j.neucir.2014.08.001 25708474

[8] Sundarakumar DK Marshall DA Keene CD Rockhill JK Margolin KA Kim LJ . Hemorrhagic collision metastasis in a cerebral arteriovenous malformation. J neurointerv Surg. (2015) 7:e34. doi: 10.1136/neurintsurg-2014-011362.rep 25261441

[9] Joret MO Starke RM Scotter J Heppner P . Metastatic choriocarcinoma induced separate simultaneous intracerebral haemorrhages: a very rare occurrence and its novel association with Klinefelter syndrome. BMJ Case Rep. (2015) 12:2015. doi: 10.1136/bcr-2015-212777 PMC4654026 26564116

[10] Vivekananda U Howard RS Matar W Phadke R . Neurological picture. Metastatic choriocarcinoma. J Neurol Neurosurg Psychiatry. (2011) 82:347–8. doi: 10.1136/jnnp.2010.220012 20884676

[11] Alkassar M Gottschling S Krenn T Graf N . Metastatic choriocarcinoma in a 17-year old boy. Klin Padiatr. (2009) 221:179. doi: 10.1055/s-0029-1220728 19437369

[12] Nishizaki T Orita T Tsuha M Wakuta Y Fujii M Ito H . Brain metastasis of testicular tumor with massive hemorrhage–report of two cases. Neurol Med Chir (Tokyo). (1991) 31:586–9. doi: 10.2176/nmc.31.586 1723173

[13] Stockton L Green E Kaur B De Winton E . Non-gestational choriocarcinoma with widespread metastases presenting with type 1 respiratory failure in a 39-year-old female: case report and review of the literature. Case Rep Oncol. (2018) 11:151–8. doi: 10.1159/000486639 PMC5903105 29681814

[14] Reilley MJ Pagliaro LC . Testicular choriocarcinoma: a rare variant that requires a unique treatment approach. Curr Oncol Rep. (2015) 17:2. doi: 10.1007/s11912-014-0430-0 25645112

[15] Richie JP . Re: A meta-analysis of the risk of boys with isolated cryptorchidism developing testicular cancer in later life. J Urol. (2013) 190:1045. doi: 10.1016/j.juro.2013.05.078 23931234

[16] Li B Cheng Q Li Z Chen J . p53 inactivation by MDM2 and MDMX negative feedback loops in testicular germ cell tumors. Cell Cycle. (2010) 9:1411–20. doi: 10.4161/cc.9.7.11255 PMC3008305 20372076

[17] Chaar A Mouabbi JA Alrajjal A Barawi M . Metastatic testicular choriocarcinoma: an unusual cause of upper gastrointestinal bleed. Cureus. (2019) 11:e5243. doi: 10.7759/cureus.5243 31565641 PMC6759043

[18] Smith ZL Werntz RP Eggener SE . Testicular cancer: epidemiology, diagnosis, and management. Med Clin North Am. (2018) 102:251–64. doi: 10.1016/j.mcna.2017.10.003 29406056

[19] Berkowitz RS Goldstein DP . Pathogenesis of gestational trophoblastic neoplasms. Pathobiol Annu. (1981) 11:391–411. 6276846

[20] Toberer F Enk A Hartschuh W Grüllich C . Testicular choriocarcinoma with cutaneous metastasis in a 19-year-old man. J Cutan Pathol. (2018) 45:535–8. doi: 10.1111/cup.13261 29665032

[21] Wanarak W Songkiet S . Intracerebral hemorrhage cause by a ruptured oncotic aneurysm from choriocarcinoma metastasis. Asian J Neurosurg. (2013) 8(1):48–50. doi: 10.4103/1793-5482.110280 23741263 PMC3667461

[22] Kidd D Plant GT Scaravilli F McCartney AC Stanford M Graham EM . Metastatic choriocarcinoma presenting as multiple intracerebral haemorrhages: the role of imaging in the elucidation of the pathology. J Neurol Neurosurg Psychiatry. (1998) 65:939–41. doi: 10.1136/jnnp.65.6.939 PMC2170418 9854978

[23] Huang CY Chen CA Hsieh CY Cheng WF . Intracerebral hemorrhage as initial presentation of gestational choriocarcinoma: a case report and literature review. Int J Gynecol Cancer. (2007) 17:1166–71. doi: 10.1111/j.1525-1438.2007.00934.x 17425677

[24] Ngan HYS Seckl MJ Berkowitz RS Xiang Y Golfier F Sekharan PK . Update on the diagnosis and management of gestational trophoblastic disease. Int J Gynaecol Obstet. (2018) 143(Suppl 2):79–85. doi: 10.1002/ijgo.12615 30306586

[25] Yokoi K Tanaka N Furukawa K Ishikawa N Seya T Horiba K . Male choriocarcinoma with metastasis to the jejunum: a case report and review of the literature. J Nippon Med Sch. (2008) 75:116–21. doi: 10.1272/jnms.75.116 18475033

[26] Zhang P Wang Y Xiong L . Gastrointestinal bleeding caused by metastatic testicular choriocarcinoma: a case report and literature review. World J Surg Oncol. (2022) 20:205. doi: 10.1186/s12957-022-02670-7 35710558 PMC9202100

[27] Yang JJ Xiang Y Yang XY Wan XR Wang RZ Ren ZY . Evaluation of emergency craniotomy for the treatment of patients with intracranial metastases of choriocarcinoma. Zhonghua Fu Chan Ke Za Zhi. (2005) 40:335–8. doi: 10.1111/j.1745-7254.2005.00209.x 15938786

[28] Suresh TN Santosh V Shastry Kolluri VR Jayakumar PN Yasha TC Mahadevan A . Intracranial haemorrhage resulting from unsuspected choriocarcinoma metastasis. Neurol India. (2001) 49:231–6. doi: 10.1179/016164101101199027 11593238

[29] Kikuchi Y Kita T Tamai S Nagata I . Choriocarcinoma with brain metastasis: report on two cases with long-term survivals. Jpn J Clin Oncol. (1990) 20:306–11. doi: 10.1093/oxfordjournals.jjco.a039405 2255107

[30] Guo J Zhong C Liu Q Xu J Zheng Y Xu S . Intracranial choriocarcinoma occurrence in males: Two cases and a review of the literature. Oncol Lett. (2013) 6:1329–32. doi: 10.3892/ol.2013.1570 PMC3813813 24179518

[31] Kohyama S Uematsu M Ishihara S Shima K Tamai S Kusano S . An experience of stereotactic radiation therapy for primary intracranial choriocarcinoma. Tumori. (2001) 87:162–5. doi: 10.1177/030089160108700310 11504371

[32] Akhavan-Sigari A Hori YS Harary PM Persad AR Kassu R Tayag A . Stereotactic radiosurgery for choriocarcinoma brain metastases: illustrative case presentation and systematic review. World Neurosurg. (2025) 194:123387. doi: 10.1016/j.wneu.2024.10.116 39491621

